# Mr. William Arthur Griffiths

**Published:** 1911-05-13

**Authors:** 


The death is reported at Micheldever of Mr. William Arthur Griffiths. He took his diploma in 1887, studied at Westminster Hospital, where he became assistant surgeon, migrated to New South Wales, and was appointed surgeon and Government medical officer to the Gunnedah Hospital. He returned to England and was appointed medical officer of the Micheldever district and surgeon to various societies.

